# Supplementary material for: Rapid turnover of life-cycle-related genes in the brown algae
Source: Genome Biol. 2019 Feb 14;20:35. doi: 10.1186/s13059-019-1630-6 (PMC6374913; doi:10.1186/s13059-019-1630-6)
Supplement: Supplementary file 3 — Protein alignments, corresponding Gblocks html file and CDS sequences for the lineage conserved gametophyte-biased genes. (ZIP 2109 kb) [file 13059_2019_1630_MOESM3_ESM.zip › Con_GA_alignments/Ec-10_001110.aa_ali.fasta-gb.htm]

Ec-10\_001110.aa.fasta.trans.aa\_ali.fasta


## Gblocks 0.91b Results

Processed file: **Ec-10\_001110.aa.fasta.trans.aa\_ali.fasta**  
Number of sequences: **2**  
Alignment assumed to be: **Protein**  
New number of positions: **286** (selected positions are underlined in blue)

```
                         10        20        30        40        50        60
                 =========+=========+=========+=========+=========+=========+
Ec-10_001110     MGSTLSKSEACMHTTWCGGGDDAYSDPPEVANDPTFP-LAEDLELNQEHVVR--SGQRLP
MSTRG.29304.1    MGSVLSKNDACM--VWCGDADDAHPEQDQTRARPVMDDDDTTVSLTGQHVAFESSAHKFA
                 ############                                                


                         70        80        90       100       110       120
                 =========+=========+=========+=========+=========+=========+
Ec-10_001110     SHSAHANLYAPAQELAPSTNEDLRIAGSGQNAPYGIPSSQRGLGSLQRKKRQ----EHRP
MSTRG.29304.1    DGPSTEQLRS-DQQLAPSTSEHLRY--TARRAQHGLPLPRRDFDSVEIHVSTQLLDSGES
                             ############       ##############               


                        130       140       150       160       170       180
                 =========+=========+=========+=========+=========+=========+
Ec-10_001110     DSLRAAASLVEEPLTPAPMTTDRLLQREYMGDGTEDGVDEGLSSFFHPERDELMEDIEWL
MSTRG.29304.1    ESRQERTPLMAELLTPPTIPRALLMQTSP-EDAIQDGGVEGLKGFFHSDGDELVEDTGWS
                  #########################     #############################


                        190       200       210       220       230       240
                 =========+=========+=========+=========+=========+=========+
Ec-10_001110     PTKRGPSLAALLQRVEYLGNVDMALSEVRSMQEQDNRGEGVVRSKVLERFLLLMGPEGPG
MSTRG.29304.1    SPKRGPSLAELLRRVEYLGNVDLALSEVRSLQEGEDEVEAMIRRTVLERFLLVMGPGGPG
                 ############################################################


                        250       260       270       280       290       300
                 =========+=========+=========+=========+=========+=========+
Ec-10_001110     LAVKKYQRSMTFGTGGSERGKWADRTIRFAWNEMDSLEDGTPLGGAIVWNSKRLFPREQR
MSTRG.29304.1    LMVKKCSRRKKNGTGGSERQKWAARTIRFEPNGMDCLSDGTPLGGAIVWNSKRLFPREKR
                 ############################################################


                        310       320       330       340       350       360
                 =========+=========+=========+=========+=========+=========+
Ec-10_001110     EVRIADIAKVEHSGNCVWMTADGEGRLGFETSSNMDARFLCRSIDFLVSATVQHRTHSGG
MSTRG.29304.1    GVDITDIAKVEHNGKSLWVTAEGDRRLGFETTSNLDARFLCRSIDFLVSAAAHHKKTVGR
                 ############################################################


                        370       380
                 =========+=========+========
Ec-10_001110     DGSKAYSLTSTGNND-APAVPASY---L
MSTRG.29304.1    PCSNASSLTSGGTNFGAYPSLDGFLPGG
                 ##############
```

```
Parameters used
Minimum Number Of Sequences For A Conserved Position: 2
Minimum Number Of Sequences For A Flanking Position: 2
Maximum Number Of Contiguous Nonconserved Positions: 8
Minimum Length Of A Block: 10
Allowed Gap Positions: None
Use Similarity Matrices: Yes
```

```
Flank positions of the 5 selected block(s)
Flanks: [1  12]  [73  84]  [92  105]  [122  146]  [152  374]  

New number of positions in Ec-10_001110.aa.fasta.trans.aa_ali.fasta-gb:  286  (73% of the original 388 positions)
```
